# Remarks on Syphilis

**Published:** 1840-12-12

**Authors:** W. Poyntell Johnston


### MEDICAL EXAMINER.

DEVOTED TO MEDICINE, SURGERY, AND THE COLLATERAL SCIENCES.

No. 50.] PHILADELPHIA, SATURDAY, DECEMBER 12, 1840. [Vol. III.

REMARKS ON SYPHILIS. *Extracted from a Lecture delivered before the Philadelphia Medical Society.* By W. Poyntell Johnston, M. D.

——There are three general and important questions to be considered in reference to syphilis, each of which groups around it all those subsidiary points which are necessary to constitute the entire study of the disease. These questions bear upon the origin—the nature—and the treatment of the affection.

1. What is the origin of syphilis? Whence was it first derived?

The solution of this question is *practically* so unimportant, that I am only induced to refer to it at all, because popular opinion has unjustly fixed upon our own country the opprobrium of having originated the disease, and distributed it, through the followers of Columbus, to the civilized world. An unprejudiced examination of this question, however, based upon a comparison of dates and circumstances, leads to the following conclusions:

1. That we have strong presumptive evidence that the disease existed, not only in its primary, but also in its secondary forms, prior to the fifteenth century, that is, *before the discovery of America.* This is not absolutely demonstrable, or of course the question would be settled. But when we find Hippocrates speaking of suppuration of the genital organs, in connexion with ulcerations of the mouth, and Galen, in his Commentary, appending to this description, that the sweats and evaporations in the genital organs occasion sometimes superficial pustules—sometime pruriginous ulcerations which by their acrimony disorganise the skin—*destroy it like eating ulcers*, (I quote his words,) it is impossible not to acknowledge that if these were not the effects of syphilis, they bore a most marvellous resemblance to them.

But 2. Suppose we lay this of one side as insusceptible of proof, and acknowledge that syphilis first appeared in 1493 or 1494, and was first seen in the southern part of Italy, in or about Naples, even under this view of the question, the amount of probabilities is in absolute opposition to its American origin.

1. Because the writers contemporary with the first appearance of the disease, and who first saw and described it as a scourge till then unknown, and one which evidently excited their strongest interest, never for a moment entertained the slightest idea of its foreign origin and importation, but sought an explanation of its appearance, sometimes in natural, more generally in supernatural influences.

It is thus in turn ascribed by them—to a miasm generated by an inundation of the Appenines—to the influence of the stars (astrology being then greatly in vogue)—and to divine interposition to check and to punish the licentiousness of the age, but never to a contagion imported from a foreign land. It was not till a later period, when the details connected with the invasion of the affection were lost, and when men's minds were only occupied with the startling facts, that the previous age had witnessed the discovery of a new world and the introduction of a new pest into the old one, that the two were associated together as cause and effect, and thus, probably, a simple coincidence has been made to stamp our country with a stigma, which, however unmerited, will, with the mass of mankind, remain attached to it for ever.

2. Because, by collating dates and facts, it can be rendered clear that syphilis could not have been imported by the followers of Columbus on his return from his first voyage. The proofs here are so strong that even the advocates of the American origin of syphilis are compelled to abandon this ground, and fall back upon the second voyage of Columbus, in which a greater number of followers participated. The first of these returned to Europe in 1494, and are said, while affected with this disease, to have accompanied Gonsalvez of Cordova to Naples, where it is universally acknowledged that syphilis first displayed itself. Now, leaving out of consideration the circumstance that even if some of the followers of Columbus were to be found in the ranks of Gonsalvez, of which there is no proof, yet in none of the districts travelled to reach Naples did they leave any trace of the affection behind them, we have a positive refutation of this opinion in the fact that Gonsalvez of Cordova did not cross from Sicily to Naples till several months after the departure of Charles VIII, that is, till after the existence of syphilis in his army.

3. Now add to these arguments that although supposed to be endemic in St. Domingo at the period of the visit of Columbus, it has never been known to exist there endemically since—while more recent travellers in other countries make it endemic in Guinea, Java, Ethiopia, China, and elsewhere, and I think we may fairly assume that, however obscure the origin of syphilis may be, there is no one rational ground upon which to charge the opprobrium of generating it upon America, but, on the contrary the immense mass of probabilities is opposed to such a supposition.*

We come now to a more essential point in the study of syphilis, viz., the question of its intimate nature—one of a practical importance very different from that just discussed—one which, since the experiments by inoculation, particularly those of Ricord, has, for the first time, admitted of solution, and consequently it is from this period alone that a rational plan of treatment can date. Here we are fortunately no longer embarrassed by speculations or contradictory statements. The labours of recent experimenters have enriched us with a mass—not of opinions, but of facts, from which we are enabled to deduce principles as positive as any which exist in the whole domain of the science. The questions which formerly agitated the profession—as whether syphilis and gonorrhœa were distinct diseases or only varieties of the same affection—whether differences in the character of chancres were attributable to a difference in the virus producing them, &c. &c., are now completely set at rest; they will all be alluded to more at large in their proper place, but at present, in order to economise time, and render the study of the nature of syphilis more instructive, it is essential to follow some system. The plan I shall adopt in treating this subject will be—first, to announce the laws which are deducible from the phenomena of inoculation, and then to group around each the facts which constitute the material proofs of the truth of these laws.

As inoculation is applicable only to the first stage of syphilis, it is of course with primary syphilis alone that we are at present occupied.

By primary syphilis is to be understood a disease characterised by the existence of a chancre. The chancre may exist in various situations, under different forms, and in different stages of advancement; it may be simple, or complicated with other affections; but under all circumstances its existence is essential—the chancre constitutes the primary syphilis.

A chancre is a specific sore, produced only by the contact of the specific matter from a chancre, and secreting itself an analogous matter, which, during a certain stage of its existence, is capable of reproducing a chancre by contact. This law is absolute, a chancre alone can produce a chancre. If a small portion of the matter of chancre be placed upon a healthy part, under circumstances favourable to contagion, as its introduction beneath the epidermis, its application to an abraded surface or a mucous membrane, its contact with the subcutaneous cellular tissue, a chancre will in each case be the result, although the circumstances attending its development will be somewhat different. Thus, if introduced beneath the epidermis, the following, according to Ricord, will be the phenomena, presented in the order in which they will occur. During the first twenty-four hours, the point pricked, as after the introduction of vaccine matter, becomes red; from the second to the third day a slight tumefaction is observable, and a small papula appears, surrounded by a red areola. From the third to the fourth day the epidermis, raised by a liquid more or less opaque, assumes often a vesicular form, offering at its summit a black point which is probably due to the dessication of blood from the prick; from the fourth to the fifth day the morbid secretion augments, becomes purulent, assumes a pustulous form, offering at its apex the umbilicated appearance of small pox. At this period the areola, the extent and intensity of which had been continually increasing, frequently begins to fade or diminish; but from the fifth day the subjacent tissues which generally had not undergone any change, or were merely slightly œdematous, may infiltrate or harden by the effusion of a plastic lymph. giving to the touch a feeling of hardness and elasticity almost cartilaginous. Finally, from the sixth day, the pus generally thickens, the pustules wrinkle, and soon scabs begin to form; if these are not rubbed off, their base enlarges, and they elevate themselves by stratified layers to assume the form of a truncated cone with a depressed summit; if the scabs are detached or fall, there is found beneath an ulcer, which, reposing upon an indurated base, (of which we have spoken as liable to occur from the fifth day,) presents an excavation, the depth of which is measured by the entire thickness of the skin, and which is covered by a whitish-gray surface, difficult to detach. The result of the inoculation is, therefore, a chancre passing through the stages of vesicle and pustule. When the virus is applied to other tissues than the skin, the result is equally a chancre, but where an epidermis or epithelium are wanting, the vesicular and pustular stages must be wanting also; while, when the matter has been introduced into the subcutaneous cellular tissue, its irritation will cause the development of the chancre to be accompanied by a phlegmonous inflammation. There is then a difference in the phenomena preceding the full development of chancre in these various modes of inoculation, but the final result is always the same,—*Whenever the specific virus of chancre is placed in contact with a healthy tissue, under circumstances favourable to contagion, a chancre will be produced.*

Again, the remainder of the proposition with which I set out, that *nothing but the matter of chancre will produce chancre*, is equally demonstrable by inoculation. It is in vain that you will inoculate the matter of gonorrhœa, of leucorrhœa, of gonorrhœal ophthalmia, or the matter of any of the *secondary* forms of syphilis itself; an inflammation may indeed be the result, which may terminate in an ulcer, but this ulcer, however much it may vary in its character, will

* For a detailed history of syphilis, see Dict. des Sciences Medicales.

never be a chancre; will never be capable of reproduction. The latter sore may then be regarded as specific.

But is a chancre always identical, or does it present varieties? Chancres manifestly vary in their appearance, situation, and gravity. Is this the result of a difference in the acrimony, or in the nature of the matter from which they were derived? Inoculation shows that it is not,—that these varieties are attributable to two causes: 1st, to a difference in the anatomical structure of the parts which are the seats of the disease; and, 2d, to a difference in the general health of the individuals affected. Inoculate with matter from the same chancre different structures in the same individual, as for example, the prepuce and the gland, and the chancres of inoculation *will differ* in appearance. Inoculate the same structure, say the inside of the thigh, in the same individual, with matter taken from different chancres, simple and phagædenic, mild and acrid, the ulcers resulting *will not differ*. And, again, inoculate different individuals with matter from the same sore, and the sores produced may present *every variety*. There is, then, no difference in the virus; but the chancres produced will be either simple or phagædenic, varying with the general health of the individual affected, but will bear no relation to the character of the sores from whence they were derived; in a person of sound constitution they will be simple, while they only become phagædenic under two opposite circumstances—excessive local inflammation, or great general prostration. It is alone by producing one or other of these circumstances, that general diseases modify the sore. The character of the general disease exerts no influence. Even secondary syphilis, the result of a previous chancre, makes no change either in the liability to a new infection, or in the character and march of the new chancre when contracted. I am aware that this is contrary to the opinion of some writers, who go so far as to assert that syphilis existing constitutionally, will render ulcers accidentally produced by other causes syphilitic. They have probably, however, mistaken coincidences for effects; for in all the cases cited, the syphilitic nature of the ulcer, when it existed, can be traced to direct contagion.

But it is not at every stage of its existence that the matter of chancre is inoculable: three distinct periods may be assigned to its march. The first constitutes its forming stage; the second embraces that period during which, after having attained full maturity, it remains stationary; and the third is the period of reparation, when, having lost its specific nature, and become a simple ulcer, it is advancing towards cicatrization.

In the first of these stages the chancre is not only inoculable, but is purely a local disease; in the second the matter remains still contagious; while in the third all power of reproduction is entirely lost.

The fact that a chancre is only inoculable during its two first stages, is directly demonstrable; but the proofs that during its first period it is purely a local affection, although unequivocal, are less absolute than those I have hitherto relied upon. It has been seen that induration is often an accompaniment of chancre; the period of its invasion is indefinite, but the results of inoculation show that it never can occur before the fifth day of the disease. Now no cases are recorded of the existence of the constitutional effects of syphilis following a chancre when the primary sore was clearly healed prior to this period; in fact, from an examination of a large number of cases of secondary syphilis in the Venereal Hospital of Paris, it is shown that in no case had the chancre a duration of less than ten or fifteen days, and that they generally had continued for a much longer period, and in all cases where a satisfactory account could be obtained, induration had accompanied them. Here is at once a strong inducement to connect the period of induration with the period at which the disease becomes constitutional and capable of producing its general effects. Abundant evidence confirmatory of this view, (which cannot now be examined in detail,) is to be found in the difference of treatment required before and after this period. We may assume, for the time, that chancre is at first a purely local disease, capable, however, of generalizing itself at variable periods after the fifth day, and that the general infection is commonly preceded by a local induration.

Chancre is a disease peculiar to the human species. All the efforts made by Hunter, Trumbull, Ricord, and others, to produce it in animals, have entirely failed, and yet no precautions were omitted. The matter employed was tested simultaneously in man, and produced the specific pustule. The animals selected were of every variety, and no possible road or method by which the virus could be introduced was neglected, but all in vain.

It has been already stated that the matter of chancre alone can produce chancre—it may be objected to this, that the matter of gonorrhœa and that from a suppurated bubo not unfrequently are capable, by inoculation, of producing the same specific sore. This apparent contradiction is easily reconciled. Chancre is confined to no one part of the system. The organs of generation are not its exclusive seat. Wherever the matter is deposited under favourable circumstances there we will find the local disease; witness the inoculation of the fingers of accoucheurs,&c., and wherever the local disease exists it is capable of becoming constitutional.

The genital organs are only more frequently the original seat of the disease from their being more frequently exposed to infection under circumstances the most favourable—but the power of inoculation of the contagious matter is not limited to an abraded surface or to the follicles of the prepuce and gland, it may be deposited in the canal of the urethra, and there produce its specific sore with all its essential characters and properties. This is not a supposition, but the assertion of a fact demonstrated by post mortem examination in cases where death has occurred from some other cause during the existence of the chancre. The distance to which the specific matter may be carried into the urethra is not limited to the vicinity of its orifice, where the resulting chancre is visible and defined, but it may be transported to any depth. I have seen one case in the Venereal Hospital of Paris, and the specimen is still preserved by M. Ricord, and open for inspection, where a primary chancre, the result of inoculation, existed on the internal surface of the bladder itself. The fact that a chancre may exist in the interior of the urethra being once proven, the explanation of the occasional inoculability of the matter of gonorrhœa, which would otherwise militate against the idea I have supported of the unity of chancre, is at once clear—the matter of simple gonorrhœa is no more inoculable than ordinary pus, but when this matter is mingled with the specific virus of chancre, provided it does not dilute it too much, this virus still retains its contagious properties, and is capable of producing its characteristic ulcer.

Again, the fact that the discharge from a suppurated bubo will occasionally by inoculation give rise to a chancre is not less easily explained on the grounds which I have adopted. A bubo may be caused by the existence of a chancre in the penis in two distinct ways—the ulcer may merely irritate the extremity of a lymphatic vessel, producing a corresponding irritation and subsequent inflammation of the nearest lymphatic ganglion, terminating in suppuration, and giving rise to an ordinary phlegmonous abscess, the contents of which are pure pus which is not inoculable; or, on the other hand, the absorbents may take up some particles of the specific virus and transport them in substance to the nearest ganglion, where they will be arrested, and retaining their power of self-production, will determine an abscess containing a contagious matter analagous to that from which it originated, and possessing all its properties.

In the first instance we have a sympathetic bubo, the result of irritation, not inoculable.

In the second, a symptomatic bubo, the result of inoculation, constituting eventually a large chancre, which isperfectly inoculable itself.

We are now prepared to draw a distinction founded upon real differences between venereal and syphilitic affections. The former include all those diseases the mere result of impure coition. The latter are constituted by chancre and its consequences alone.

To resume, then, we may consider syphilis as a specific disease, strongly analagous to variola; a pustular disease capable of unlimited reproduction by inoculation, purely local while it retains its pustular form, but frequently contaminating the whole system when this period has passed, and the resulting ulcer has been neglected.

An attempt to consider the varied symptoms by which a constitutional syphilis, the result of this neglect, betrays itself, would carry me beyond the limits allotted to a subject which is offered for discussion. I wish simply to attract attention to the fact that it is at first local, and only subsequently generalises itself, for upon this fact are founded the indications now generally adopted for its treatment in those different stages.

The contradictory opinions of practitioners, in reference to treatment of syphilis—dividing them into mercurialists and non-mercurialists—can be reconciled only by admitting this view of the subject. Like all other controversies, upon points of science, where intelligent men are diametrically opposed, and engage their feelings in the discussion, it has resulted that, each are right to a certain degree, and both are wrong in being too exclusive. While the disease is local, a general treatment would be superfluous; and mercury, at this stage, is even counter-indicated,—it is not curative, for the sore heals as rapidly without as with its employment,—it is not prophylactic, for secondary symptoms are as frequent after its exhibition, as when it has been neglected; and there is strong ground for supposing that it is frequently absolutely injurious, by superadding to the other secondary symptoms—one among the most serious—exostosis.

But when the disease becomes constitutional, its whole character is changed, and the indications for its treatment change with it. Now, local applications, if exclusively relied upon, are incompetent to cure—they may remove the induration, the index of a constitutional taint, but the constitutional taint itself is beyond their reach. General remedies alone can destroy it, and these vary with the stage of the disease—if exhibiting itself by secondary symptoms, mercury is, without exception, the most efficient agent—if by tertiary symptoms, which mark a profound alteration of the whole system, which depend upon a syphilitic cachexia, mercury is again counter-indicated as aggravating the affection, by adding to the deterioration of the fluids.

To resume, in the local stage mercury is of no service—in the secondary symptoms, in nine cases out of ten, it acts as a specific, and in the tertiary symptoms it is clearly injurious.

